# Platelet-Rich Plasma in Bone Regeneration: Engineering the Delivery for Improved Clinical Efficacy

**DOI:** 10.1155/2014/392398

**Published:** 2014-06-23

**Authors:** Isaac A. Rodriguez, Emily A. Growney Kalaf, Gary L. Bowlin, Scott A. Sell

**Affiliations:** ^1^Department of Biomedical Engineering, The University of Memphis and Joint University of Memphis-UTHSC-Memphis Biomedical Engineering Program, 330 Engineering Technology, Memphis, TN 38152, USA; ^2^Department of Biomedical Engineering, Parks College of Engineering, Aviation, and Technology, Saint Louis University, 3507 Lindell Boulevard, St. Louis, MO 63103, USA

## Abstract

Human bone is a tissue with a fairly remarkable inherent capacity for regeneration; however, this regenerative capacity has its limitations, and defects larger than a critical size lack the ability to spontaneously heal. As such, the development and clinical translation of effective bone regeneration modalities are paramount. One regenerative medicine approach that is beginning to gain momentum in the clinical setting is the use of platelet-rich plasma (PRP). PRP therapy is essentially a method for concentrating platelets and their intrinsic growth factors to stimulate and accelerate a healing response. While PRP has shown some efficacy in both *in vitro* and *in vivo* scenarios, to date its use and delivery have not been optimized for bone regeneration. Issues remain with the effective delivery of the platelet-derived growth factors to a localized site of injury, the activation and temporal release of the growth factors, and the rate of growth factor clearance. This review will briefly describe the physiological principles behind PRP use and then discuss how engineering its method of delivery may ultimately impact its ability to successfully translate to widespread clinical use.

## 1. Introduction

Normal healthy bone has the ability to spontaneously regenerate during remodeling or after minor injury. However, if the defect site exceeds a critical size (such that the bone will not spontaneously heal during the animal's or patient's lifetime), bone grafting is required to regenerate new tissue [1, 2]. Common bone graft biomaterials include autografts (a patient's own bone), allografts (human cadaver bone), xenografts (animal bone), and synthetic biomaterials [3–7]. Of these, autografts are used as the current standard since they are osteogenic, osteoconductive, and osteoinductive [3]. Although autografts produce satisfactory results, they carry the risk of donor site morbidity and are limited in availability. With auto-, allo-, and xenografts, each having their own unique set of disadvantages, synthetic biomaterials are emerging as potentially viable substitutes for bone regeneration, considering that they satisfy requirements such as being biocompatible, biodegradable, and bioactive. From 1999 to 2009, the sales of bone grafts and bone-graft substitutes in the US alone increased from 0.3 to 1.5 billion dollars with increased spending on platelet concentrators, bone substitutes, bone morphogenetic proteins, and more [8]. Platelet-rich plasma (PRP, a platelet concentrate) can be used alone or in combination with scaffolds and biomolecules as an alternative bone graft substitute.

PRP is a concentration of platelets in blood plasma. In a healthy human, average circulating platelet counts are approximately 200,000 platelets/*μ*L. Clinically, PRP is typically administered at a severalfold increase over that baseline concentration [9, 10]. The interest in concentrated platelets is derived from their early role in the normal healing response. Platelets contain more than 300 biologically active molecules which are released upon activation and subsequently influence the tissue regeneration process [11, 12]. Activated platelet-derived factors serve as messengers and regulators that influence a variety of cell-cell and cell-extracellular matrix (ECM) interactions [13–15]. In addition, it has been shown that a linear relationship exists between platelet concentrations and the concentration of available cytokines. This is attractive to tissue engineering and regenerative medicine since increasing the number of platelets available in a defect/injury site will increase the amount of bioactive cytokines capable of stimulating and accelerating the repair process [16].

Platelet alpha and dense granules release an array of bioactive molecules upon activation (Table 1) [9, 11, 14, 17–19]. Activated PRP contains platelet-derived growth factor (PDGF), transforming growth factor-*β* (TGF-*β*), vascular endothelial growth factor (VEGF), epidermal growth factor (EGF), fibroblast growth factor (FGF), and others [9, 11, 13, 14, 17–20]. PRP also contains a number of macrophage and monocyte mediators and a variety of interleukins (IL) capable of mediating inflammation [9, 11, 13, 18, 21]. Furthermore, the plasma component of PRP contains the proteins fibrinogen, albumin, several immunoglobulins, and more [11, 22–24]. Several of these bioactive molecules play a significant role in bone remodeling. A list of these factors and their functions is detailed in Table 2.

The clinical use of PRP has expanded into treatment of multiple tissues, albeit with varying degrees of effectiveness. PRP therapy (in various delivery methods) has been implemented to stimulate tissue regeneration in bone, cartilage, skin, ligament, tendon, muscle, and more. This therapy typically involves an autologous blood draw and centrifugation to separate and obtain the platelet concentrate. PRP is then activated (commonly by CaCl_2_ and/or thrombin) and applied to the defect/injury site. However, it has been shown that thrombin as a clotting agent to form a platelet gel can result in rapid activation of platelets and ultimately a mass release of growth factors (70% released within 10 minutes and nearly 100% released within 1 hour) [9]. These growth factors, which undergo a burst release, are cleared before they can have any stimulatory effects on cells [25]. When platelet gels are formed using CaCl_2_, growth factor release can be slowed. CaCl_2_ activates and clots PRP by forming autogenous thrombin from prothrombin leading to the eventual formation of a loose fibrin matrix that will release growth factors over 7 days [9]. As bone regeneration is a lengthy process (adequate strength typically restored within 3–6 months), there is an obvious need for effective delivery vehicles capable of the sustained release of PRP-derived factors over an extended period of time to maximize their regenerative potential. This review details the regenerative advantages of PRP and examines various techniques and scaffolding options for the sustained delivery of PRP-derived growth factors to diseased or damaged bone.

## 2. Liquid (Unactivated)

Historically, PRP in a purely liquid form has rarely been used to treat bony defects; it was most often activated by thrombin or CaCl_2_ to form a platelet gel for therapeutic use. However, PRP is also readily activated in the presence of collagen type I, and because of the rapid release of growth factors after platelet activation, it can be beneficial to use a direct injection method in cases where the releasate would form immediately upon injection and contact with the area of injury, particularly with injured or damaged soft tissues such as ligaments and tendons [26]. With regard to using unactivated liquid PRP for bone regeneration, few studies have been performed, most likely due to the challenges associated with delivery and retention of the unactivated form.

### 2.1. Liquid PRP Alone

Nikolidakis et al. investigated the effects of liquid PRP immersion on goat trabecular bone implants [27, 28]. In this study, liquid PRP or activated PRP gel was applied to cylindrical titanium oral implants, half of which were coated with a thin layer of calcium phosphate (CaP). The CaP surface configuration for implants has been shown in previous studies to enhance bone response in the initial healing period [29] and has been proved as such in this study; however, incorporation of PRP on the implants had a dichotomic affect: bone response to the CaP-coated implants was not significantly improved for either forms of PRP, potentially due to the degradative effect of CaP coatings on PRP [30], but liquid PRP appeared to have a significant improvement on bone apposition to the implants in the early healing stages before implantation. In a similar manner, Yun et al. studied titanium implant osseointegration supplemented with bone marrow-derived mesenchymal stem cells (BMMSCs), porous hydroxyapatite (HA), and liquid PRP in canine trabecular bone [31]. Histometric analysis of the various combinations of the implant supplements in the intrabony defects yielded no statistical significance with regard to bone density and bone-to-implant contact, but the results suggested that the addition of liquid PRP contributed to increased bone healing. HA and PRP have been researched previous to Yun's work; the addition of PRP to a HA/collagen type I bead matrix within a polytetrafluoroethylene (PTFE) graft attached to rabbit iliac crests was performed by Chang et al. in an attempt to create an artificial autogenous bone graft [32]. Liquid PRP was injected into the grafts weekly, with blank controls (no HA/collagen type I) and PRP controls. As stated previously, liquid PRP is activated in the presence of collagen type I, and therefore it may be presumed that, upon injection, the PRP was activated and it expressed the growth factors necessary for ingrowth of tissue. Radiological results showed significantly increased mineralization in the group with the beads and weekly PRP addition. Histological results also supported this finding. However, a mild inflammatory response was also found in the HA + collagen type I + PRP group as well as a “creeping substitution of the implant by the ingrowth of granulation and macrophage tissues,” suggesting a foreign body reaction. As with any healing process, a short-term inflammatory response is expected. Long-term regeneration can still be achieved, depending on the progression of inflammation. Though this inflammatory response was mild, particularly with respect to the regenerative effect of the bead system and PRP addition, it should be noted that the exact cause of the effects, whether from the PTFE graft or from the contents of the graft, was not determined [32].

### 2.2. Liquid PRP Blended with Demineralized Bone Matrix

In the case of demineralized bone matrix (DBM), inactivated PRP had a significantly increased effect on osteoinductivity compared to activated PRP according to research by Han et al. [33]. In this study on athymic rats, osteogenesis, chondrogenesis, and proliferation of osteosarcoma and bone marrow stromal cells were significantly enhanced by nonactivated PRP, and were inhibited by activated PRP. The authors speculated that platelet activators other than thrombin could activate the PRP over time, reducing the inhibitory activity of PRP activated in high doses as shown by other studies [34–36]. DBM contains the collagen necessary to activate PRP, but in amounts small enough to create a slower, more sustained activation and release of PRP growth factors than the thrombin activation [37]. In all of the publications combining a liquid PRP with DBM, the platelet concentrations were either approaching or above the standard PRP platelet count of 1 million platelets per *μ*L [10], or approximately 5 times above baseline (Table 3). As is common in PRP literature, there were a range of published platelet concentrations and this deviation should be taken into account in regard to comparing studies for future research.

## 3. Gels

As previously described, the activation of PRP results in the formation of a platelet gel. PRP can be activated to form a gel by the addition of CaCl_2_, autologous or allogeneic thrombin, CaCl_2_ + thrombin, Ca-gluconate + thrombin, CaCl_2_ + batroxobin, or through contact with exposed collagen [38–42]. The most commonly used activators for PRP include 10% CaCl_2_ and thrombin, alone or in combination. The use of CaCl_2_ induces clotting by natural thrombin activation. This clotting cascade is slower, which provides the option of injecting the platelet concentrate before gelation. Immediate clotting (3–5 seconds) can be induced by the addition of CaCl_2_ + thrombin. Although this platelet substance cannot be injected, it is immediately available for use as a gel [43]. Each has their own advantage, depending on the intended application. In 2004, Waters and Roberts conducted a study which isolated factors that were important in manufacturing a consistent gel to better understand the gelation process and how different consistencies could be obtained. It was determined that ultimately, the gelation of PRP was independent of platelet count and fibrinogen concentration [44].

Characterization of platelet gels is critical to the understanding of the mechanisms behind their effects. As mentioned, the use of PRP provides many advantages. One such advantage which is not often mentioned, particularly with platelet gels, is its antibacterial properties. An* in vitro* study conducted by Bielecki et al. found that autologous platelet gels inhibited the growth of various bacteria responsible for wound, bone, and chronic ulcer infections, as well as common hospital-acquired infections of surgical wounds and infections associated with indwelling medical devices [45]. Evidence in the literature suggests that platelets play multiple roles within the antimicrobial defense system. Some of these include navigation toward the inflammatory chemoattractant N-Met-Leu-Phe, expression of immunoglobulin-G Fc receptors and for C3a/C5a complement fragments, and the ability to produce antimicrobial oxygen metabolites including superoxide, hydrogen peroxide, and hydroxyl free radicals. In addition, platelets directly interact with microorganisms, actively participate in antibody-dependent cell cytotoxicity against microbial pathogens, and contribute to the clearance of pathogens from the bloodstream [46–48]. Ultimately, this characteristic can improve the treatment of various infected bone injuries such as delayed healing and nonunion [45]. Within bone engineering, there are instances in which xenogeneic materials (such as grafts or minerals) are used to enhance bone repair. This proves more difficult with platelet gels since there are large intraspecies variations. It was found that human PRP contained higher growth factor concentrations per platelet when compared to rat and goat PRP. The one commonality was that TGF-*β*1 was the most abundant growth factor in the PRP of all three species. These results suggest that although PRP contains osteoinductive growth factors, they are most likely species related.* In vitro* experiments support this since rat bone marrow cells cultured on human, rat, and goat PRP gels performed best on rat PRP gels by stimulating initial growth and bone differentiation [38]. Further characterization of platelet gels details that PRP growth factors can be successfully incorporated and released while remaining active and having positive effects on bone healing. Specifically, platelet gel releasate has been shown to increase proliferation, collagen synthesis, mineralization, and alkaline phosphatase (ALP) of osteoblasts* in vitro*. Furthermore, a dose dependent administration of platelet gel releasate can affect cell behavior [39]. In addition to enhanced proliferation in the presence of platelet gel releasate, stromal stems cells (conditioned with platelet gel releasate) can mineralize the ECM once releasate is removed [49].

### 3.1. Platelet Gels Alone

As previously mentioned, the activation of PRP forms an antibacterial platelet gel consisting of multiple active growth factors. This gel can be used alone or in combination with various components as a bone regenerative substitute. Both* in vivo* and clinical studies show convincingly positive results when platelet gel alone is used as an alternative bone graft [50–53]. Two separate* in vivo *rat studies revealed that PRP gel improved diabetic fracture healing (to equal normal healing) as well as histological healing, healing quality, and bone strength in fracture healing [40, 54]. A two-year, 60-patient, molar defect randomized comparative clinical study conducted by Ogundipe et al. recorded enhanced and faster bone healing as well as reduced swelling, pain, and trismus for patients treated with PRP gel [55]. In addition to oral and maxillofacial applications, platelet gels also have potential for the regeneration of long bone. Rupreht et al. conducted a 50-patient randomized clinical study where PRP gel applied after autograft positioning for ACL surgery enhanced cortical bone formation encircling the tibial tunnel at 2.5 and 6 months [42]. Although a 13-month, 22-patient clinical study conducted by Galasso et al. showed no improvement in nonunion long-bone regeneration using platelet gels, the platelet gel group reported fewer complications [41]. Clinically, platelet gels alone have a high success rate. In the instance where platelet gels behave similar to standard grafting procedures, these scaffolds still prove advantageous by reducing the need for autologous grafting, ultimately resulting in fewer complications.

### 3.2. Platelet Gels Supplemented with Cells

Comparing acellular and cellular therapy has been an area of interest for many years. Some believe that implanting a scaffold without cells to recruit the host's precursor or tissue-specific cells is the best method for tissue regeneration. Others feel that implanting a scaffold precultured with precursor or tissue-specific cells is more promising for tissue regeneration. The previous section discussed the successes of platelet gels alone, while this section will briefly explore platelet gels supplemented with precursor or further differentiated bone-specific cells. As of today, most cell supplemented platelet gel studies have been conducted* in vivo* using rat, mouse, rabbit, and dog models. Several of these* in vivo* studies concluded that platelet gel combined with BMMSCs enhances bone regeneration when compared to controls [56–59]. It has also been shown that proliferation of rat bone marrow cells incorporated within a platelet gel can be enhanced in a dose dependent manner. This suggests that a high platelet concentration in combination with osteoblastic cells within the platelet gel could accelerate the formation of new bone,* in vivo* [60]. Clinically, the use of mesenchymal stem cells (MSCs) in a platelet gel has potential for periodontal applications by reducing bone defect depth, probing depth, bleeding (upon probing), and tooth mobility [61]. Aside from various MSCs, few other cell types have been combined with platelet gels. The thought here is that the environmental and scaffold cues will drive these autologous osteoprogenitor cells down an osteoblastic (bone forming) lineage. One other cell type that has been studied in combination with platelet gels is MC3T3-E1 osteoblastic cells. Goto et al. conducted this* in vivo* subcutaneous ectopic experiment using a mouse model. They reported the formation of mineralized tissue and expression of osteocalcin and collagen type I [62]. Overall, the combination of platelet gels with osteoblast progenitor cells can serve as a viable clinical alternative to autografts. This is achieved by providing a scaffolding complex that delivers the appropriate cues to promote osteoblastic differentiation of the supplemented cells.

### 3.3. Platelet Gels Blended with Autologous and/or Allogeneic Bone

**Figure 1:**
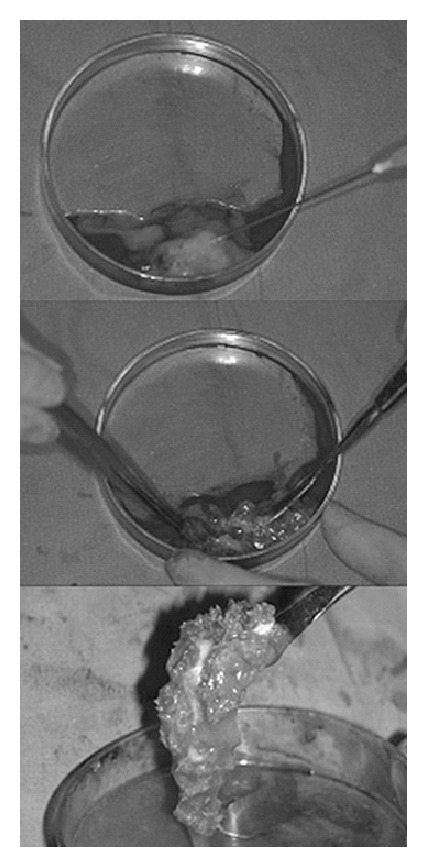
Preparation of autologous bone-platelet gel. Autologous bone particles mixed with PRP followed by activation. This gel-consistency scaffold can be easily handled [64]. Reprinted with permission from Elsevier.

Considering that autografts are the standard procedure for clinical bone grafting, it is intuitive to examine the potential of autologous bone in combination with platelet gels (Figure 1). Few* in vivo* studies have been conducted to explore this combination. Such study confirmed that autologous cancellous bone combined with an autologous platelet gel (compared to autograft alone) enhanced bone regeneration in an* in vivo* critical-size cylindrical defect (11 × 25 mm, diameter × depth) on load-bearing long bones of minipigs [63]. Clinically, the majority of autologous platelet gel and bone graft experiments focus on oral and maxillofacial bone regenerations with much success, short- and long-term [64–66]. Not only have autologous bone-platelet gels enhanced bone formation, but they are also associated with enhanced healing of soft tissues around the bone and bone wound, most likely as a result of the increased level of localized growth factors [67]. While not a randomized trial, another clinical study involving 14 patients was able to quantify the advantage of using autologous bone with platelet gels compared to autograft alone. In terms of quantity of bone used, Mèndez et al. reported that 30% less cancellous bone was used in cases where autologous bone-platelet gels were implanted [68]. Although this does not completely eliminate the need for autologous harvesting, it does provide an advantageous alternative which increases bone regeneration while decreasing cost and morbidity. In some instances, such as diabetic patients, the use of autologous PRP may not be desirable. For these cases, allogeneic PRP can be used since, similar to matching blood type donations, PRP is versatile. Smrke et al. showed that allogeneic platelet gels combined with autologous cancellous bone can fully bridge bone defects in a tibia fracture of a diabetic patient [69]. This study demonstrated that, in the case where autologous PRP is not ideal or cannot be extracted, donor allogeneic PRP can still enhance bone regeneration when combined with autologous bone in a platelet gel.

Allogeneic and synthetic bone-platelet gels have also been explored as alternative bone substitutes in an attempt to reduce morbidity and the need for autologous bone harvesting.* In vivo*, the combination of platelet gel and allogeneic bone have been met with varying success from regenerating bone comparable to or better than the controls [70, 71]. Clinically, the application of allogeneic bone-platelet gels alone has not been a popular focus. Instead, allogeneic bone-platelet gels in combination with autologous and/or synthetic bone have recently demonstrated enhanced bone regeneration in dental and vertebral clinical trials [72, 73].

**Figure 2:**
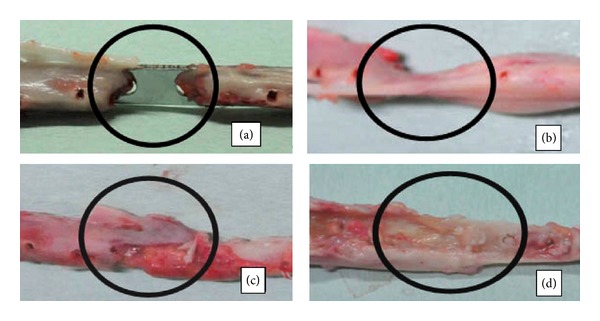
Gross examination of rabbit tibia healing after 11 weeks with (a) control group, no platelet gel or Coragraft; (b) platelet gel; (c) Coragraft; and (d) platelet gel and Coragraft [74]. Reprinted with permission from Elsevier.

Platelet gels have also been combined with artificial bone grafts to reduce the need for autologous bone grafting while comparing the synergistic effects of activated PRP with artificial bone. One such* in vivo* study conducted by Kanthan et al. demonstrated that the best bone healing based on radiological, histological, and gross findings, occurred in the platelet gel combined with artificial bone (Coragraft) group.  Figure 2 grossly depicts rabbit tibia healing over 11 weeks which ultimately demonstrates that the independent use of the platelet gel or synthetic bone alone does not promote adequate bone repair [74].

### 3.4. Platelet Gels Blended with Bone Minerals

The addition of bioactive inorganic calcium phosphates (such as CaP, HA, and tricalcium phosphate, TCP) to scaffolds creates more of a bone-like (organic-inorganic) alternative graft substitute. In addition to serving as a bone structure mimicking component, bone minerals have the ability to bind a variety of molecules, including proteins [75]. Specific to platelet gels, this characteristic proves to be advantageous in the fact that activated PRP contains a variety of growth factors and serum adhesion proteins that can potentially bind with bone minerals. This combination has the potential to enhance cellular response and ultimately bone regeneration. Although the incorporation of bone minerals with platelet gel is not commonly used clinically, oral and maxillofacial clinical studies have demonstrated the success and potential of these grafts to enhance bone formation and promote tissue healing [76–78].* In vivo* experiments, on the other hand, report various results ranging from no difference to enhanced new bone deposition when using platelet gels incorporated with bone minerals [79–83]. Although these results vary, none of the experiments (clinical or* in vivo*) report a decrease in bone regeneration when bone mineral incorporated platelet gels are used. Therefore, this combination, like other platelet gels, can still be used as alternatives to autografts since they eliminate the need for surgery to harvest autologous bone and perform comparably or better than standard techniques.

### 3.5. Platelet Gels with Various Components

Since individual or multiple components can easily be incorporated within platelet gels, several studies have experimented with various platelet gel combinations. Successful bone regeneration of sinus grafts in clinical studies supports the use of platelet gels combined with autologous bone and bovine xenograft (Bio-Oss), *β*-TCP (Cerasorb), or bioactive glass [84–86]. Recently, the use of bone morphogenetic proteins (BMPs) has also shown a positive synergistic effect when combined with platelet gels for* in vitro *and* in vivo *bone regenerations [87, 88]. On the other hand, combinations such as platelet gels with fibrin glue, human fascia lata membrane, bone marrow aspirate, or simvastatin showed no significant differences in bone healing compared to controls [89–92].Ramanathan and Carippe prepared a platelet gel where Gelfoam was soaked in PRP followed by activation which allowed the platelet gel to form throughout the Gelfoam. This was a combination of a platelet gel and a sponge (described in more detail in the following section). This clinical study reported significant bone healing for all groups with no differences between the platelet gel and the control groups [93]. Throughout the literature, there have been few instances where the addition of a composite platelet gel impaired the development of new bone. One such study recently conducted by Portela et al. reported significantly less bone matrix development and impaired osteogenesis in platelet gels enriched with leukocytes when compared to controls in a rat calvaria* in vivo *model. The hypothesis here was that since leukocytes play a role in early inflammation, they may also contribute to the enhancement of bone healing when combined with platelet-derived growth factors. The authors speculated that the lesser boney matrix deposition and the significantly lower quantities of osteocalcin observed when treated with leukocyte-enriched platelet gels could be attributed to the higher levels of TGF-*β*1 (osteoblastic maturation inhibitor) released [94].

Overall the application of various composite platelet gels has had a significant positive impact within the field of alternative bone grafting. Although there are varying results, all platelet gel implants (with the exception of leukocyte enriched platelet gels) at least performed similar to the controls, if not better. This leads to the reduction of autologous bone tissue harvesting and ultimately a decrease in surgical trauma. In addition, the gel-like consistency allows constant localized delivery of PRP growth factors and eliminates the need for implantation and removal of a membrane normally used to contain scaffold contents within the defect area.

## 4. Hydrogels and Sponges

Unlike platelet gels, where PRP is activated to form the gel structure, hydrogels and sponges require a base material (such as alginate, gelatin, or collagen) to which PRP is incorporated. Throughout the past decade, PRP incorporated hydrogels and sponges composed of alginate and gelatin have proven to be highly successful in bone regeneration. The benefit of using a hydrogel or sponge system includes the tailorable degradation of the scaffold which in turn affects the release of incorporated factors, such as PRP. This carrier system is ideal for sustained delivery and enhanced bioavailability of growth factors to the injury or defect site.

### 4.1. Alginate Hydrogels

Alginate is a well-characterized, highly investigated biopolymer that is biodegradable, biocompatible, and nonimmunogenic. Alginate hydrogels are commonly used as delivery vehicles for a variety of biomolecules and factors. By simply incorporating PRP into an alginate hydrogel, Lin et al. showed that the growth factors released from the hydrogel system stimulated the osteogenic differentiation (ALP and mineralization) of human MSCs* in vitro* [95]. Lu et al. further delved into the growth factor release kinetics of PRP incorporated alginate hydrogels, specifically, analyzing the growth factor release profile and binding to two types of alginate carriers: beads and capsules. It was determined that the growth factor (PDGF-AB, TGF-*β*1, and IGF-1) release profiles varied as a result of carrier type and that the binding of these factors to the alginate structure controlled their release. In addition to being detectable after release, growth factors remained bioactive and promoted SaOS-2 osteoblast-like cell proliferation and ALP activity* in vitro* [25]. A study performed by Huang et al. also showed that* in vitro* MSCs can be induced into an osteoblastic phenotype by a PRP encapsulated alginate hydrogel. Further* in vivo *studies where the PRP-MSCs-alginate hydrogel mixture was implanted subcutaneously in mice also resulted in enhanced ectopic bone regeneration [96]. Alginate hydrogels have proven to be successful carriers for PRP and PRP combined with cells such that the incorporated factors retain their bioactivity and have positive effects on bone regeneration after being released both* in vitro* and* in vivo*.

### 4.2. Gelatin Hydrogels/Sponges

**Figure 3:**
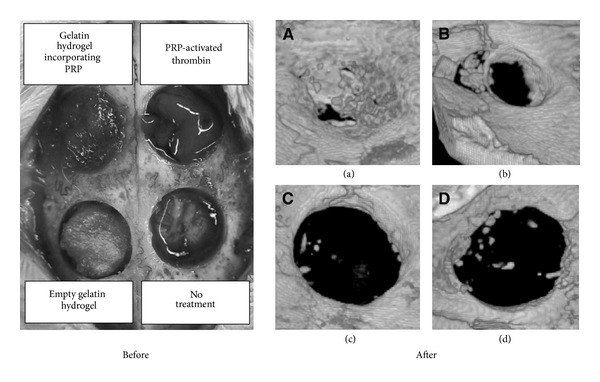
Photographs and 3D images of rabbit calvarial defects before and after 8 weeks of implantation with (a) gelatin hydrogel + PRP, (b) platelet gel, (c) gelatin hydrogel without PRP, and (d) no treatment. Complete bone regeneration was only observed in the gelatin hydrogels with PRP. Reprinted with permission from Mosby, Inc.

Gelatin (denatured collagen) is a commonly used protein for fabricating scaffolds intended for bone tissue engineering. The advantages of gelatin are that it is easier to obtain, is less expensive, and possesses similar functional groups as collagen which enhances cellular response since collagen is the main organic component of bone [97]. In the literature, when referring to gelatin, the terms “hydrogel” and “sponge” are used interchangeably depending on the author's preferences. To fabricate these hydrogels/sponges, gelatin is used as a base material dissolved in water, cooled to induce gelation, and then lyophilized to create porous hygroscopic scaffolds. The incorporation of PRP can be achieved by several methods including soaking the gelatin hydrogel/sponge in a PRP solution, adding PRP to the base gelatin solution prior to gelation, or adding PRP incorporated micelles within the scaffold [98–102]. Hokugo et al. determined that the PRP growth factors are immobilized via physicochemical interactions with the gelatin molecules within the hydrogel. This causes a release of growth factors correlating with hydrogel degradation. Two* in vivo* studies (rabbit ulna and calvarial defects) confirmed that the PRP incorporated gelatin hydrogels resulted in successful bone regeneration, which was not observed in controls [98, 99]. Microcomputed tomography (microCT) of the 5 mm calvarial defects confirmed complete bone regeneration only with the gelatin + PRP hydrogels after 8 weeks of implantation (Figure 3) [99]. Kim et al. took a novel approach with regard to cell recruitment for bone regeneration. In this study, a macrophage recruiting agent, SEW2871, of a sphignosine-1 phosphate agonist and PRP were combined in micelles and incorporated into gelatin hydrogels.* In vitro *and* in vivo* results indicated that this composite scaffold promoted a higher number of recruited macrophages when compared to hydrogels with SEW2871-micelles alone, PRP alone, or neither. It was concluded that this increase in macrophage recruitment contributed to PRP-induced bone regeneration in a rat model [100].

Gelatin hydrogels/sponges not only have the ability to incorporate proteins and growth factors, but can also incorporate nanofillers such as inorganic minerals or other nanofillers. Rodriguez et al. engineered a unique gelatin sponge incorporated with a lyophilized version of PRP (preparation rich in growth factors, PRGF), HA, and chitin whiskers (CW). The addition of PRGF significantly enhanced MG-63 osteoblast-like cell infiltration* in vitro* while the HA and CW nanofillers enhanced the mechanical integrity of the scaffold. It was also determined that sponges crosslinked during gelation degraded faster than sponges crosslinked after lyophilizing [101]. A follow-up study further investigated surface modification of gelatin + PRGF + HA + CW sponges by growing a bone-like mineral layer via simulated body fluid (SBF) mineralization. Gelatin sponge controls showed enhanced cellular attachment after the nucleation of clusters of minerals on its surface. Gelatin + PRP + HA + CW mineralized and nonmineralized sponges showed similar MG-63 cell attachment/infiltration and the ability to incorporate and successfully release PRGF growth factors. Composite mineralized sponges also degraded faster, releasing 30% of its original protein content within 21 days [102]. There are only a small number of cases which report that a gelatin + PRP sponge showed no enhancing effects for bone regeneration when compared to controls. One such study was conducted by Okamoto et al. who revealed no significant differences between the gelatin + PRP + *β*TCP sponge and the autograft control groups with respect to biomechanical stiffness or bone volume over time when implanted in an* in vivo *rat model. Although this particular gelatin + PRP sponge had no advantage, it is still an attractive alternative to autografts since it is a less invasive technique with similar results [103].

### 4.3. Collagen Sponges

Organically, bone is primarily comprised of collagen type I. As intuition leads, fabricating a scaffold from collagen would directly mimic the organic material component of bone. As a result, collagen is frequently used as the base material for sponge-like scaffolds for bone regeneration. Surprisingly, unlike gelatin, collagen sponges incorporated with PRP have more variable results in the literature, ranging from the addition of PRP enhancing to PRP having no effect on bone regeneration compared to controls. The majority of collagen sponges incorporated solely with PRP have limited potential in bone tissue engineering both* in vivo* and clinically [104–106]. Wiltfang et al. reported successful early bone regeneration in collagen + PRP sponges in an* in vivo *pig model with a critical-size defect (10 × 8 mm, diameter × depth) in the forehead region. Early bone regeneration was noticed when PRP was added to autologous bone components and not with xenogeneic bone substitutes suggesting that the combination of autologous PRP and scaffold components has more potential for bone regeneration [107]. Thus far, the PRP used within hydrogels and sponges contain the normal amount of platelets (4x to 8x baseline). In 2009, a new procedure was developed to produce 23x baseline concentrate platelets. This concentrate was incorporated within a resorbable collagen sponge, activated with calcium chloride and autologous thrombin, and clinically implanted into the sinus floor of a patient. After 5-6 months, new vital dense bone with a good trabecular pattern and connectivity was observed suggesting that an increase in platelet concentration has significant positive effects on enhancing bone regeneration [108]. In recent years, the incorporation of additional proteins, such as BMPs, to PRP collagen sponges has been explored. Again, controversial and varying results both* in vivo* and clinically are reported [109–111]. The addition of other components such as demineralized bone powder to a PRP collagen sponge has proven to be successful in an* in vivo* dog model. The combination of a collagen type I sponge embedded with demineralized bone powder, PRP, and activation factors (CaCl_2_ and thrombin) wrapped with autologous omentum and periosteum induced the high expression of osteoinductive cytokines (TGF-*β*, BMP-2, and BMP-4) in macrophages, endothelial cells, osteoblasts, osteoclasts, and the local mesenchymal tissue. The combination of these factors within a collagen sponge has the potential to produce mature trabecular bone upon implantation [112]. Although these composite PRP collagen sponges have varying results, none of them have negative effects on bone regeneration which still support the use of these sponges as an alternative bone graft substitute replacing the need for autologous bone harvesting.

### 4.4. Other Hydrogel/Sponge Materials

Aside from alginate, chondroitin sulfate succinimidyl succinate (CS-NHS) is a material in which a novel adhesive hydrogel with the capability to house multiple biomolecules can be fabricated. This unique hydrogel-biomolecule composition was recently introduced by Simson et al. who showed that a CS-NHS adhesive hydrogel is a promising delivery tool for intraoperative biologics (such as PRP and bone marrow) in orthopedic applications [113].

To veer away from the conventional gelatin/collagen sponges, Oktay et al. developed a chitosan sponge incorporated with PRP. This* in vivo* study found that PRP alone had enhancing effects on bone regeneration while the chitosan sponge had limited effects [114]. Although this study does not support the use of chitosan as a base material for bone regenerative sponges, it does provide suggestions for future direction with respect to the use (or nonuse) of different base materials for the delivery of PRP via sponges.

## 5. Nanofibers

Nanofiber scaffolds have tremendous potential in the field of tissue engineering due to their ability to replicate ECM topography on a submicron scale. These scaffolds not only are able to have controlled parameters such as fiber alignment and diameter, but also are able to exhibit high surface area-to-volume ratios and sustained passive transport and provide mechanical support [115]. However, promoting cell ingrowth and penetration into these scaffolds can be difficult; without the reservoir of soluble growth factors that are naturally found in native ECM, cells tend to remain on the surface of the scaffold [116]. Researchers have started to incorporate growth factors [117, 118], and only very recently PRP, into the nanofibers to increase this bioactivity and replicate the critical role that the ECM plays in regeneration.

### 5.1. Self-Assembled PRP Nanofibers

Yoshimi et al. and Kohgo et al. both studied the effect of PRP on the novel self-assembling nanofiber scaffold known as PuraMatrix (PM) [119, 120]. Kohgo and Yoshimi introduced the concept of incorporating dog MSCs (dMSCs) and PRP into the PM to investigate the combinatorial effects on canine bone defects caused by dental implants. Yoshimi discovered that the mature bone formed by the combination was of a very high histologic and histomorphometric quality, suggesting that the PM + PRP + MSC combination might be useful in terms of treating bone defects. Kohgo went on to discuss the bone-to-implant contact (BIC) and determined that the highest BIC upon analysis belonged to the combination group, supporting Yoshimi's suggestion regarding the usefulness of the PM combination material for bone defects.

### 5.2. Electrospun Nanofibers Coated with PRP

The delivery of bone morphogenetic protein 7 (BMP-7) matrix by a CaP coated electrospun nanofiber mesh with the addition of a PRP was investigated by Berner et al. [121]. Large bone defects were treated with 4 different combinations of CaP, BMP-7, and PRP on electrospun poly(*ε*-caprolactone) (PCL) scaffolds: an uncoated tube, a CaP tube with and without PRP, and a CaP tube with BMP-7 and PRP. For the experimental conditions, BMP-7 was loaded onto meshes after reconstitution with liquid PRP. The solution was then thrombin-activated and loaded onto the mesh tubes, creating a layer of the PRP/BMP-7 solution on the mesh surface, and inserted onto opposing ends of full thickness diaphyseal segmental rat femoral defects. Results from radiography, histology, mechanical testing, and microCT showed significantly increased bone volume and biomechanical properties for the BMP-7 + PRP mesh compared to control groups, supporting the theory that PRP can enhance BMP-7 delivery via mesh tube and bone defect regeneration.

### 5.3. Electrospun PRGF Fibers

Two articles have been published regarding the electrospinning and subsequent formation of PRP fibers. Wolfe et al. and Sell et al. published research findings on electrospinning activated and lyophilized PRP into fibrous scaffolds [115, 122]. In the article by Sell et al. PRGF was incorporated into silk, PGA, and PCL and the resulting scaffolds were characterized via SEM and protein release quantification as well as through human adipose derived stem cells (hADSCs) interaction and macrophage proliferation. Results of the study showed that the incorporation of PRGF into the various scaffolds had a significant positive influence with regard to the bioactivity, as proliferation and chemotaxis of the cell lines had significant increases over control. Wolfe et al. characterized the effects of varying concentrations of electrospun pure PRGF scaffolds on protein release, fiber diameter, and cell interaction. In doing so, this study demonstrated the feasibility of a pure PRP-based scaffold that is stable, exhibits a sustained protein release, and promotes rapid cellular infiltration* in vitro*. Cell infiltration into the scaffold increased as the PRGF concentration increased for both the hMSCs and the hADSCs used in the study, potentially due to the increased concentration of chemotactic growth factors and the increased void space found in the PRGF scaffold with increased concentration. Fiber diameter supported previous research regarding the linearity of the polymer concentration, showing increased diameter with higher concentrations [123, 124].

Of the five authors researching PRP involvement with nanofibers, only two mentioned the concentration of platelets in the PRP solution (Table 4). The two authors that noted the concentration did not quantify their platelets in house. Because of this lack of information, it is unclear whether the results of the study would be applicable to other studies in regard to protocol or scaffold manufacturing.

## 6. Bioactive Glass and PRP

The combination of bioactive glass and PRP is not commonly studied for bone regeneration. However, two independent* in vitro* studies confirmed enhanced bone regeneration using PRP and bioactive glass. The first study conducted by Dutra et al. used bioactive glass foams (produced by the sol-gel process) with and without PRP to regenerate premolar defects in dogs. Glass foams both with and without PRP showed an increase of bone thickness and histological bone formation compared to controls (no biomaterial). The PRP associated group showed a thicker bone area and a more mature bone formation than bioactive glass foams without PRP. These results show that bioactive glass foams associated with PRP can maintain the thickness of the alveolar ridge and improve bone formation [125]. The second study combined bioactive borate glass (BG) with PRP to regenerate bone in a diaphyseal rabbit model. After 12 weeks of implantation, histology and microCT revealed that the PRP-treated BG group yielded enhanced bone formation [126]. Although bioactive glasses are not a material popularly associated with PRP, these combined scaffolds prove to be effective in repairing bone defects and warrant further investigation.

## 7. Conclusions

Based upon the published literature, particularly the large collection of* in vitro* work demonstrating improved cellular response, there is little doubt that the growth factor milieu contained within PRP has the potential to be highly beneficial to bone regeneration. The primary issues with clinical PRP use currently stem from the variability in its use. This variability includes differences in methods to create and activate PRP, platelet concentration, and delivery, making it impossible to directly compare clinical studies. Regardless of methodology, another issue for the use of PRP in bone defects is the challenge of retaining PRP growth factors at the defect site in a physiologically active state. Going forward, the ability to reduce the above mentioned variability and to provide a sustained release of PRP biomolecules may be critical to expedited healing of bony defects. Building upon the body of early* in vitro* work performed on PRP, recent years have shown a marked increase in its utilization in a number of animal models and human trials. This collection of studies is truly translational, taking advances in drug delivery, biomaterials, and tissue engineering and applying them to the efficacious delivery of PRP growth factors to stimulate bone regeneration. While to date there has not been a definitive delivery method that has distanced itself from the others in terms of efficacy, the increase in number and diversity of approaches seems to indicate that the clinical potential of PRP in treating bone defects has been recognized. It is up to the ongoing and future research in areas such as PRP hydrogels, sponges, and nanofiber scaffold fabrication to fully realize that potential and create a clinically successful formulation and delivery method.

## Figures and Tables

**Table 1:** Some PRP bioactive molecules and their physiologic roles [127]. Reprinted with permission from John Wiley and Sons.

General category	Specific molecules	Physiologic role
Adhesive proteins	Fibrinogen, fibronectin, vitronectin, thrombospondin-1, von Willebrand factor, and laminin-8	Cell contact interactions, cellular adhesion, chemotaxis, ECM composition, and clotting

Clotting factors and associated proteins	Factor V, factor XI, protein S, antithrombin, and tissue factor pathway inhibitor	Thrombin activation and its regulation, eventual fibrin clot formation

Fibrinolytic factors and associated proteins	Plasminogen, plasminogen activator inhibitor, *α*-2 antiplasmin, and thrombin-activatable fibrinolysis inhibitor	Plasmin production and regulation

Proteases and antiproteases	Tissue inhibitor of metalloproteases 1-4 (TIMP 1-4), MMP-1, -2, -4, and -9, and *α*-1 antitrypsin	Regulation of matrix degradation, regulation of cellular behavior, and so forth

Growth factors, cytokines, and chemokines	TGF-*β*, PDGF, insulin like growth factors (IGF) I and II, FGF, EGF, VEGF, ECGF, KGF, GM-CSF, hepatocyte GF, TNF*α*, RANTES, IL-8, IL-1*β*, and BMP-2, -4, and -6	Chemotaxis, cell proliferation and differentiation, promotion of ECM production, regulation of inflammation, and angiogenesis

Antimicrobial proteins	Thrombocidins and kinocidins	Bactericidal and fungicidal properties

Membrane glycoproteins	*α*IIb*β*3, *α*v*β*3, CD-40 ligand, P-selectin, tissue factor, PECAM-1, tissue factor, and CD63	Platelet aggregation and adhesion, inflammation, and platelet-leukocyte interactions

Lipids	Sphingosine-1-phosphate, HETEs, thromboxane B_2_, prostaglandin F_2_ *α*, leukotriene B_4_, and lipoxin A_4_	Inflammation modulation, cell migration and proliferation, and so forth

Basic proteins and others	Platelet factor 4, *β*-thromboglobulin, endostatins, connective tissue activating peptide III, chondroitin-4 sulfate, albumin, and immunoglobulins G and M	Regulation of endothelial cell chemotaxis and angiogenesis, vascular modeling, platelet activation, and so forth

**Table 2:** Role of PRP components in bone remodeling.

PDGF	Mesenchymal stem cell (MSC) and progenitor cell recruitment, proliferation, migration, and osteogenic differentiation. Osteoblast proliferation and ECM ossification [25, 128, 129]

TGF-*β*	MSC recruitment and differentiation. Increased production of collagen and mineral matrix. Inhibits osteoclast formation and bone resorption [128–130]

TGF-*β*1	MSC recruitment, proliferation, and osteogenic differentiation [25]

IGF-1	Stimulates bone formation via cellular proliferation, differentiation, and synthesis of Type I collagen [25, 128, 129]

IL-1, IL-6, TNF-*α*	Promotes early responses of bone repair, endochondral bone formation, and bone remodeling [129, 131]

Basic FGF	MSC growth and differentiation. Osteoblast proliferation [132]

Fibronectin, vitronectin	Enhances formation of focal adhesions by osteoblasts, osteoblast migration [133]

VEGF	Promotes angiogenesis and endochondral ossification [25]

VGF, platelet microparticles	Promotes angiogenesis [131]

**Table 3:** Summary of studies which used PRP in its inactive form.

Authors	Study	Platelet concentration	Results
Nikolidakis et al. (2006) [27], Nikolidakis et al. (2008) [28]	CaP + Ti implants	8–12 × 10^5^/*μ*L	Significantly increased bone contact percentage with Ti + PRP (liquid) implants Significantly higher endosteal bone formation length in CaP + PRP (gel) coated implants
Yun et al. (2013) [31]	HA + BMMSCs + Ti implants	Adjusted to 1 × 10^6^/*μ*L	No significance found
Chang et al. (2009) [32]	HA/collagen type I bone graft formation	9.25–12.5 × 10^5^/*μ*L	Significant bone tissue in HA/collagen + PRP (liquid injection)
Han et al. (2009) [33]	DBM + active versus inactive PRP	8x over baseline∗	PRP (liquid) significantly increased osteoconductivity of DBM

Note (∗) that the study by Han et al. [33] did not give exact platelet counts.

**Table 4:** Summary of studies that used PRP via incorporating into nanofiber matrices.

Authors	Study	Platelet concentration	Results
Berner et al. (2012) [121]	Allogenic PRP + BMP-7 + CaP coated electrospun PCL	Unknown	Significantly increased bone volume and biomechanical properties
Sell et al. (2011) [122]	PRGF scaffold characteristics and effects on human macrophages and ADSCs	955 × 10^3^/*μ*L (quantified elsewhere)	Increased macrophage chemotaxis, increased proliferation, and infiltration of ADSCs. Sustained protein release discovered
Wolfe et al. (2011) [115]	PRGF scaffold characteristics and effects on human ADSCs and hSMCs	955 × 10^3^/*μ*L (quantified elsewhere)	Cell integration into the scaffold after 3 days
Yoshimi et al. (2009) [119]	Self-assembling peptide “PuraMatrix” nanofiber scaffolds + PRP + dMSCs	Unknown	PRP alone did not promote bone regeneration, but it was facilitated with addition to dMSCs + PM
Kohgo et al. (2011) [120]	Osseointegration of dental implants with PM + PRP + dMSCs	Unknown	PM + dMSCs + PRP showed significant osseointegration over other groups
